# Stigma in attention deficit hyperactivity disorder

**DOI:** 10.1007/s12402-012-0085-3

**Published:** 2012-07-08

**Authors:** Anna K. Mueller, Anselm B. M. Fuermaier, Janneke Koerts, Lara Tucha

**Affiliations:** Department of Clinical and Developmental Neuropsychology, Faculty of Behavioural and Social Sciences, University of Groningen, Grote Kruisstraat 2/1, 9712 TS Groningen, The Netherlands

**Keywords:** Stigma, Stigmatization, Attitudes, ADHD, Children, Adults

## Abstract

Attention deficit hyperactivity disorder (ADHD) is a frequently diagnosed disorder in child- and adulthood with a high impact affecting multiple facets of social life. Therefore, patients suffering from ADHD are at high risk to be confronted with stigma, prejudices, and discrimination. A review of the empirical research in the field of ADHD with regard to stigma was performed. The findings of investigations in this field were clustered in different categories, including stigma in children with ADHD, stigma in adults with ADHD, stigma in relatives or in people close to a patient with ADHD, and the influence of stigma on authorities’ attitudes toward patients with ADHD. Variables identified to contribute to stigma in ADHD are public’s uncertainty concerning the reliability/validity of an ADHD diagnosis and the related diagnostic assessment, public’s perceived dangerousness of individuals with ADHD, socio-demographical factors as age, gender, and ethnicity of the respondent or the target individual with ADHD, stigmatization of ADHD treatment, for example public’s skepticism toward ADHD medication and disclosure of diagnostic status as well as medication status of the individual with ADHD. The contribution of stigma associated with ADHD can be conceptualized as an underestimated risk factor, affecting treatment adherence, treatment efficacy, symptom aggravation, life satisfaction, and mentally well-being of individuals affected by ADHD. Public as well as health professionals’ concepts about ADHD are highly diverse, setting individuals with an ADHD diagnosis at greater risk to get stigmatized.

## Introduction to stigma in mental disorders and attention deficit hyperactivity disorder

During the last 10 years, the number of studies examining the impact of stigma, prejudice, and discriminating behavior on the mental health and life satisfaction of people at risk for, or already diagnosed with a mental disorder (Brohan et al. 2010) increased considerably. In general, stigma reflects the expression of a discrediting stereotype deriving from falsely assumed associations between a group of people and unfavorable characteristics, attributes, and/or behaviors (Demaio 2006). Stigma, as an overall construct, is conceptualized as a modifiable but chronic and culturally formed environmental stressor (Zelst 2009; Corrigan and Shapiro 2010). Stigma utilization and stigma perception can be described as a complex interplay of cognitive, affective, and behavioral features foremost noticed and expressed in social interactions (Goffman 1997). Three qualities of stigma can be differentiated, including *public stigma*, *self*-*stigma* (Corrigan and Shapiro 2010), and *courtesy stigma* (Goffman 1963). According to Corrigan and Shapiro (2010), *public stigma* can be noticed when a large population collaboratively accepts discrediting stereotypes about out-group members or more cursory, individuals from groups that are perceived to differ in physical, behavioral, or other intrinsic characteristics. Corrigan and Calabrese (2001) as well as Forbes and Schmader (2010) added that symptoms or the pure label of a mental disorder increase the individual’s risk to be set apart from society and to become a victim of *public stigma*. Fausett (2004) suggested that overtly devaluating minority groups might be a consequence of modern ideals of autonomy and independence, which are widely perceived to be limited in people suffering from mental illnesses (Fabrega 1990). Consequently, *public stigma* frequently results in *self*-*stigma*. *Self*-*stigma* as described by Fabrega (1990) is the individual’s internalization of a “new degraded identity” that negatively impacts on the individual’s social functioning and its quality of life. Accordingly, individuals’ loss of social- or work-related status is one of the likely consequences of stigma (Fabrega 1990). *Courtesy stigma* represents the phenomenon that family members or people close to a stigmatized person get negatively judged due to their mere association with the stigmatized target (Tuchman 1996; Kendall and Hatton 2002; Norvilitis et al. 2002; Koro-Ljungberg and Bussing 2009; dosReis et al. 2010). Recent investigations on stigma in mental disorders emphasize that stigma may even initiate a transition from formerly light deviant symptoms to full psychiatric, thus clinical significant disorders (Rüsch et al. 2005; Zelst 2009). This cascade is at least partly evoked by strengthening the patient’s disorder perception and restraining the individual from disclosing its symptoms to others (Demaio 2006; Zelst 2009). With regard to attention deficit hyperactivity disorder (ADHD), it appears that the existence of stigma and its impact on the diagnosed individual’s life is highly under-investigated. This is surprising considering the disorder’s vulnerability of eliciting stigmatizing perceptions in the public. Goffman (1963) assumed disorders with a highly unknown and arbitrary etiology or with symptoms that are believed to be under the individual’s control are more likely to trigger public stigmatization. Indeed, the few experimental studies examining healthy participants’ reactions toward individuals displaying ADHD symptoms showed that participants highly discredited their diagnosed counterparts’ behavior. Nearly, all of the healthy participants quoted ADHD symptoms to be childish and socially inappropriate (Canu and Carlson 2003; Stroes et al. 2003). Furthermore, the presentation of behaviors which are prototypical for ADHD (videotapes) increased both tendencies of peer rejection and feelings of hostility in undiagnosed peers (Paulson et al. 2005). This finding demonstrated that the emotional state of undiagnosed individuals’ can be altered by the mere exposure to the disorders’ symptoms. Moreover, ADHD`s association with social norm violation and the society’s tendency to accuse affected individuals of being unwilling to fit into the social system makes the diagnosed person likely to face consequences of stigma. In line with this, Slopen et al. (2007) pointed out that antisocial behavior and dangerousness of individuals with mental illnesses were one of the main topics of the American press when reporting on psychiatric disorders. ADHD and its association with a range of educational, emotional as well as social adjustment problems might therefore be very likely to become the focus of public debates concerning the possible dangerousness of people diagnosed with ADHD. Prejudices about symptom etiology (Clarke 1997) further strengthen misperceptions that either the individuals by themselves or their environments are to be blamed for their condition (e.g., ADHD is caused by excessive sugar consumption, poor parenting, or unfavorably behaviors during pregnancy, such as smoking or alcohol consumption) (Clarke 1997). Finally, general mistrust and the increase in public debates about the immediate and long-term effects of ADHD medication (Stine 1994) may further contribute to the stigmatization of individuals suffering from ADHD.

## Method

A review of English published literature of several databases (PsycInfo, SocIndex, Web of Science, PubMed) on the key terms “ADHD” and “stigma” revealed a total of 33 articles that were closely related to stigma in patients with ADHD. Investigations on stigma in ADHD cite from 1994 to 2011 with the majority of studies being conducted in the first decade of the twenty-first century. Further elaboration on themes as social representations, rejection, and perceptions associated with ADHD led to another 5 articles, discussing the relevance of stigma in ADHD. The literature overview will start with studies dealing with *self*-*stigma* in ADHD-diagnosed children, followed by studies assessing stigmatizing attitudes of unaffected children toward peers suffering from ADHD and stigma affecting the classroom situation of children with ADHD. It will be continued with studies focusing on stigma associated with an ADHD diagnosis in adulthood as expressed by undiagnosed individuals and will close with studies dealing with courtesy stigma due to raising children with ADHD.

### Stigma perception in children with ADHD

The first article considering the impact of stigma on children diagnosed with ADHD was published by Stine (1994) who mentioned the relevance of stigma in the context of children’s noncompliance to stimulant drug treatment. Stine (1994) assumed that stigmatizing prejudices toward ADHD medication ultimately increases patients’ noncompliance to therapy and causes patients to be more cautious in disclosing their condition to others. Despite former and current research underlining the efficacy of ADHD medication (Hinshaw 2006; Toplak et al. 2008) with around 80 % of all ADHD-diagnosed children receiving medication to reduce their symptoms (Clarke 1997), misperceptions of ADHD medication are frequent in children with ADHD as well as in their families and friends (Stine 1994). For example, stigmatizing beliefs about long-term and immediate consequences of ADHD medication such as the risk of becoming addicted or being no longer under the control of ones’ senses has been mentioned. Furthermore, taking medication to improve ADHD symptoms might carry the risk to induce feelings of being different from peers. As revealed by Clarke (1997), ADHD-diagnosed children expressed stigmatizing beliefs concerning negative side effects of ADHD medication that clearly contributed to discomfort and dysfunctional self-perceptions (low self-esteem). Harpur et al. (2008) assessed reactions of both children with ADHD and their parents toward ADHD medication by making use of the Southampton ADHD Medication Behavior and Attitudes Scale. Results indicated that ADHD-diagnosed children associated the intake of ADHD medication predominantly with costs rather than with benefits, whereas parents reported the exact opposite. Costs related to ADHD medication were conceptualized as decreased levels of pleasure and activity as well as negative effects of medication on personality as reported by children. Levels of reported costs were significantly related to both children’s perception of stigma and to resistance to ADHD medication intake (Harpur et al. 2008). These findings indicate that a reduction in children’s perceived levels of stigma might have a beneficial impact on the child’s willingness to accept ADHD medication and by this general treatment adherence. According to the literature overview by Davis-Berman and Pestello (2010), the impact of stigma related to stimulant medication on the self-esteem of children with ADHD is still inconsistent. These authors suggested that lowered self-esteem might stem from the individual’s perception of being dependent on medication intake to function adequately in everyday life situations. However, the study by Kendall and Shelton (2003) illustrated that it remains difficult to conclude whether negative self-perceptions of children with ADHD stem from the mere diagnosis of ADHD, from medication, negative expressions of others, or a combined effect of all of these factors. Also, Davis-Berman and Pestello (2010) found that children with ADHD do not necessarily associate lowered levels of self-esteem with stigma related to medication intake. Their participants complained about not having met their own and parental expectancies with regard to academic achievements and linked their failure perception frequently to the fact of being diagnosed with ADHD. Therefore, the authors concluded that the individuals’ blaming of their ADHD for the negative consequences they come to know in life is a sign of patients’ internalized self-stigma.

Furthermore, medication disclosure has not been found to lead to increased reports of peer rejections or social rejection by others (Sandberg 2008; Davis-Berman and Pestello 2010; Singh et al. 2010). In line with this, Singh et al. (2010) proposed that stigma is more likely to arise from ADHD-specific symptoms than from medication intake itself. As can be seen in campus students’ ratings concerning the increased consumption of ADHD medication, ADHD medication intake of undiagnosed peers was not associated with any stigma (DeSantis et al. 2008). Advokat (2010) reported that up to 8 % of undergraduate students in the United States apply for ADHD medication without reliable symptom presentation. Furthermore, a considerable amount of individuals with ADHD reported that they have been approached by fellow students to sell them surpluses of their ADHD medication (Davis-Berman and Pestello 2010). Attempts to get access to ADHD medication were not only motivated by academic interests but also by recreational intentions, such as being able to party excessively. Davis-Berman and Pestello (2010) proposed that stigma associated with stimulant medication might be inconsistent across age groups. Students, apparently, grow up in an atmosphere in which taking stimulants is propagated to be generally accepted, whereas older generations might have a more cautious opinion regarding psychotropics. Instrumentalization of ADHD medication by patients or misuse of ADHD medication by healthy individuals, however, may significantly contribute to stigmatizing ideas, ultimately undermining ADHD as a clinical condition. Therefore, it is not surprising that prejudiced peer interactions of accusing individuals with ADHD to take medication for social and/or academic benefits or comments questioning the legitimacy of ADHD as representing a real disorder were frequently reported (Davis-Berman and Pestello 2010).

Alarming high numbers of children with ADHD stated personality changes due to medication intake (Davis-Berman and Pestello 2010; Harpur et al. 2008), such as being no longer oneself or being less interested in social interactions when being on medication. These experiences might contribute negatively to the emotional and personal development of individuals with ADHD and by this lead to isolation, depriving peers, and others of valuable contacts with individuals with ADHD, which in the long-run might result in stigmatizing attitudes. This is further promoted by biased media reports citing considerable side effects of ADHD medication. According to Stine (1994) and Schmitz et al. (2003), selective coverage of negative press about ADHD medication contributes to the formation of stigmatizing attitudes toward individuals with ADHD. dosReis et al. (2010) mentioned parental concerns induced by highly negative loaded media statements about ADHD medication. A quarter of parents participating on the study stated very explicit ideas, such as ADHD medication causing addiction, turning children into zombie-like creatures or devastating the individual’s career. However, studies tracing stigma perception of ADHD-diagnosed children are not restricted to lowered self-esteem due to medication intake but also focused on the question whether children with ADHD hold less stigmatizing attitudes toward peers with ADHD than undiagnosed children. Accordingly, Coleman et al. (2009) investigated response styles toward a fictional peer depicted in a short story suffering from one of three conditions (asthma, depression, and ADHD). They revealed that children with ADHD hold negative beliefs about their own condition, which might indicate signs of self-stigma or a general lack of knowledge. Participants who were diagnosed with either asthma or depression attributed the fictional child’s ADHD condition more often to parenting and/or substance abuse than children with ADHD. These findings indicate that being diagnosed with a mental disorder does not prevent individuals from holding misperceptions about their own as well as other’s clinical conditions and consequently does not prevent them from engaging in stigmatizing beliefs. Maladaptive cognitions and behavior arising from stigma perceptions have been shown to affect emotional well-being of adolescents at high risk for ADHD and were further associated with clinical symptoms of depression, maladjustment, and lowered self-esteem (Kellison et al. 2010).

In order to attenuate misperceptions concerning ADHD, Stine (1994) recommended more sensitivity of health professionals when communicating the diagnosis and possible treatment options to patients and their families. Burch (2004) proposed that stigmatizing attitudes toward and perceptions about ADHD can be reduced by increasing the knowledge about ADHD etiology and the disorder’s impact on patients’ life. This is in line with a review about effect studies of stigma campaigns (Corrigan and Penn 2004) showing that their success seemed to depend not only on the implemented strategy (i.e., *protest, education, and contact*) but also on the characteristics of the addressed audience, the stigmatized group, and interactional factors arising from the complexity of social processes (Corrigan and Penn 2004).

### Stigmatization of children with ADHD by unaffected peers

Former research indicated that children suffering from various diseases such as obesities, physical impairments/deviances, or learning disabilities seem to have an increased risk to get confronted with stigmatizing attitudes from unaffected peers (Crystal et al. 1999; Musher-Eizenman et al. 2004). Moreover, discriminating perceptions of peers due to race or ethnicity have been reported (McGlothlin et al. 2005). Tuchman (1996) hypothesized that children’s well-being might be disproportionally more determined by the degree of encountered peer acceptance than adult’s sentiments toward them. Furthermore, children’s norms do not have to match the norms of adults, placing children at higher risk to experience negative consequences from peer rejection than from rejection by adults (Tuchman 1996).

As Hoza et al. (2005) indicated, ADHD-diagnosed children are overall less favored as friends by peers and acknowledged as highly disturbing in the class environment, making it likely that prejudices associated with the diagnostic label increase. With respect to children’s approval of an ADHD-diagnosed peer, Liffick (1999) assessed 5 dimensions of stigma (blame, sympathy, anger, help, and acceptance) in two different groups of school children toward fictional characters depicted in short stories (vignettes) that were either diagnosed with ADHD, displayed high levels of aggressiveness, were in a wheelchair or diagnosed with Down syndrome. The results revealed that healthy children judged a peer with a diagnosis of ADHD or an aggressive animus more negatively compared to the remaining conditions. These findings are supported by further research showing that stigmatizing attitudes toward ADHD-diagnosed peers can be already objectified in children (Law et al. 2007; Coleman et al. 2009). Furthermore, the results demonstrated that stigma toward mental disorders or behavioral deviances, which are perceived to be under the individual’s control, elicited more negative perceptions in unaffected peers than medical or physical conditions (Adler and Wahl 1998; Fausett 2004; Law et al. 2007).

While some studies revealed that girls are more liberal in judging others (Zahn-Waxler and Smith 1992; Cohen et al. 1997), others failed to find an effect of gender on the degree of stigma toward a fictitious character with ADHD as presented in vignettes (Law et al. 2007; Liffick 1999). In the study of Law et al. (2007), the majority of participants (80 %) associated the described disruptive behavior of the vignette’s character with being male, indicating that boys might be more easily associated with deviant behavior than girls. However, such observations may also lead to the suggestion that externalizing behaviors in girls would be less accepted by peers in general. This idea is supported by the finding that negative peer ratings were more likely if the ADHD-associated deviant behavior was displayed by a female fictitious character (Fausett 2004).

It has been found that adding a diagnostic label of ADHD to a description of a child with behavioral problems (e.g., vignette) did not reveal any further explanation of the overall negative ratings of participants. Law et al. (2007) therefore concluded that it is more likely that the sample’s levels of disapproval can be attributed to the externalizing behavior of the vignette’s character per se and is not enhanced by the label “ADHD.” This is supported by Cornett-Ruiz and Hendricks (1993) who showed that prototypical ADHD behavior was a stronger predictor of children’s peer ratings concerning the diagnosed child’s future success, the affectional state toward the diagnosed child, and predictions concerning the diagnosed child’s accomplishment of a hand-written essay than adding a diagnostic label to the ADHD-diagnosed child as depicted on a video. However, it has to be considered that not all children might have heard of ADHD before. For example, in the study of Law et al. (2007), only 8 % of a total of 63 % of those children who stated being familiar to peers with comparable behavioral deviance ever heard about ADHD before.

Walker et al. (2008) also investigated peer reactions toward three types of vignettes. Vignette types were, “Michael a boy suffering from ADHD,” “Michael suffering from depression,” and finally a control condition with “Michael being diagnosed with asthma.” Against the researchers’ expectations, respondents assigned the most negative stigmatizing ratings to the fictional character displaying symptoms of depression followed by the ADHD condition. According to Walker et al. (2008), the respondents’ threat perception elicited by the vignette-child’s behavior was a significant source of stigma utilization toward peers. A phenomenon that was also noticed in former research (Martin et al. 2007; Pescosolido 2007; Pescosolido et al. 2007) showing that the degree of perceived threat is a potent moderator in heightening avoidance tendencies toward children suffering from mental disorders.

Correlation analysis between expressed social distance and potential causative attributions indicated that peers who rated the vignette-child’s disorders to stem from parental failure, substance abuse, or the individual’s own failure (low effort) were also more likely to prefer to distance from the target (Coleman et al. 2009). Based on attribution theories that predict stigmatizing attitudes to increase in response to attitudes of blaming the victim for its condition, Coleman et al. (2009) interpret these correlations to reflect an “underlying construct of individualistic, moralistic and blaming view” of the participants. Sandberg (2008) examined whether explicit disclosure of medication status impacts on stigmatizing attitudes toward peers with ADHD by measuring reactions of boys (7–11 years of age) toward age-matched playmates who were either introduced as “having ADHD symptoms,” “taking medication at school,” or characterized by “suffering from ADHD symptoms and being medicated.” In contrast to previous studies (Harris et al. 1992), this examination did not elicit stigmatizing judgments of participants due to the manipulation of introductory information. Somewhat unexpected, Sandberg (2008) even found an enhancing effect on the unaffected peers’ willingness to socially interact with a peer by giving reference to the diagnostic and medication status of the prospective playmates. Behavioral recordings indicated that the undiagnosed boys even actively animated the peer with ADHD to work on the common task. However, playmates, who got actually treated with medication focused more stringent on the task, were more good-natured and more talkative toward their unaffected peer, compared to boys with ADHD from which medication status was unknown. It is therefore difficult to decide whether the positive peer interactions were measured due to actual absence of prejudices or because of the affected child’s level of approach behavior. As outlined above, Harris et al. (1992) also found an effect on unaffected boys’ sentiments toward prospect playmates by the information used when introducing a child. Harris et al. (1992), however, did not give reference to any diagnostic label but introduced the boys’ prospective playmate as being highly disruptive and that a “hard time” can be expected when playing with the peer. Results showed that introducing unaffected peers to suffer from behavioral problems impacts negatively on peers’ ratings even though actual deviant behavior was not present. During interactive play, peers who were made believe that their play partner was suffering from behavioral problems were less likely to positively reward their partners’ achievement. As a consequence, children who were labeled as suffering from behavioral problems were less inclined to attribute successful accomplishment of the task to their own competence, indicating a detrimental effect on children’s self-efficacy by negative believes others hold about their social functioning. The differences between the findings of Sandberg (2008) and Harris et al. (1992) most likely resulted from methodological differences. Sandberg (2008) missed to check for levels of familiarity of the participating boys with the diagnostic label of ADHD and boys’ knowledge about ADHD medication in general. Therefore, unaffected peers’ attitudes might not be primed by stigmatizing expectancies. Harris et al. (1992) manipulation on the other hand gave reference to direct aversive behavior, making it more obvious to the unaffected peer what has to be expected. Consequently, future studies measuring actual peer interaction as an index of stigma should control for children’s knowledge and expectancies about ADHD in order to find out whether different introductions of children with ADHD to peers cause different behavioral effects.

### Public stigma toward children with ADHD

Martin et al. (2007), Pescosolido (2007), Pescosolido et al. (2007, 2008) as well as McLeod et al. (2007) analyzed empirical data of the “National Stigma Study-Children” which studied the attitudes of American adults toward children suffering from either a mental or physical condition (symptoms of ADHD, symptoms of depression, and asthma) without giving explicit reference to the child’s diagnostic status. Martin et al. (2007) revealed that adults preferred the highest levels of social distance toward illustrations of children that displayed clinically significant symptoms of ADHD or major depression. Moreover, the degree of rejection toward children displaying ADHD symptoms was two to three times higher compared to the remaining conditions. Around 25 % of the respondents did not want “their child to make friends with a child with ADHD” and around 20 % expressed clearly that they do not want to engage with a child presenting behavior typically seen in ADHD. Furthermore, around 50 % of the surveyed adult participants attached a stigma to help-seeking behavior (psychotherapy or medication) in general.

Factors moderating participants’ sentiments toward children with ADHD included blaming the individual or its family for misbehaving, age of the fictitious character (with older, especially male children with ADHD being more avoided), and characteristics of the adult participants (with female, married and better-educated individuals seeking more distance from a child with ADHD). In general, better-educated participants were more cautious in ascribing deviant behavior to be signs of a mental disorder (Pescosolido et al. 2008). Furthermore, cultural background has been found to affect levels of stigmatization. Compared to white adults, adults from African–American origin presented with more lack of information concerning ADHD were less likely to accept ADHD to be a medical condition and appeared to be more mistrusting toward teaching instructors or general school staff if faced with a child’s deviant behavior (Pescosolido 2007). Furthermore, participants were less able to identify ADHD compared to depression and more than half of participants failed to identify ADHD pathology in children. Accepting ADHD as representing a real disorder obviously impacted on respondents’ opinions about treatment necessity. Similar to patient’s own expressed fears (Stine 1994; Clarke 1997; Harpur et al. 2008), adults worried that treatment for mental disorders will lead to long-lasting negative social consequences (Pescosolido 2007). In this context, Pescosolido et al. (2008) suggested that stigmatizing conceptions about stimulant medication may adversely affect patients engaging in help-seeking behavior, therapy adherence, and ultimately therapy efficiency. Moreover, participants reported that they would less likely turn to close relatives, friends, community hospitals, and psychiatrists than to teachers, medical doctors, and mental health professionals, if they would be confronted with ADHD in their own child (Pescosolido et al. 2008). Another effect on disclosing tendencies was found to be related to the age of the participant, with older participants indicating that they would be less likely turn to family and friends. This is in accordance with the findings of Krendl et al. (2006) who reported an increased vulnerability of older individuals to respond more conservative toward individuals at risk for stigmatization. The largest gaps in knowledge about ADHD have been reported in men, participants from non-white ethnical backgrounds, and older people (McLeod et al. 2007). Therefore, public health and education campaigns have been proposed to address the lack of knowledge and misperceptions regarding ADHD (McLeod et al. 2007). In this context, Olfson et al. (2003) assumed that an increased public awareness of ADHD and more information formulated to correct misperceptions might ultimately assist the diagnosed individuals to seek out treatment by reducing general public stigma.

Focusing on ADHD-related issues in different ethnicities, Kendall and Hatton (2002) reported that differences in access to ADHD-related health care for children seem to exist in nearly one-fourth of the cases due to the racial background of the child, with Caucasian children being more likely to get treated for ADHD than African–American children. The authors further assume stigma related to an ADHD diagnosis to be one of the mechanisms causing differences in quality and quantity of access to health services and observable substandards of medical supplies in African–American populations in the United States. While emphasizing the unintentional character of the apparent disparities, Kendall and Hatton (2002) suggested that harmful differences may arise from automatically elicited stigmatizing associations of minorities and signs of mental disorders. Accordingly, when asking the public about typical symptoms of ADHD, disproportionally high degrees of negative traits are mentioned that are similar to features prejudicially accredited to young African–Americans in general (i.e., increased rates of deviant behavior). For example, symptoms like oppositional and violent behavior are as frequently mentioned to be features of ADHD as they are believed to be characteristically for juvenile African–Americans. Furthermore, it appears that there is a tendency to accuse non-white and less privileged families more frequently to raise children with behavioral and adjustment problems than white, middle-class families. Consequently, Kendall and Hatton (2002) hypothesized that the public will be more inclined to accuse ADHD symptoms in African–American to be caused by poor parenthood, lower intellectual functioning, substance abuse, violent behavior, and/or poverty than ADHD symptoms in white Americans.

### Stigmatization by authorities

It has been shown that teachers’ expectancies concerning their pupils’ achievements formed on the basis of outer appearance and manner influences children’s actual school performance (Rosenthal and Jacobson 1968). As expectancies can be conceptualized as cognitive precursors of attitudes, it can be assumed that if they are marked by prejudices they easily may turn into stigmatization. Attitudes of teachers might be of particular importance for children with ADHD considering that difficulties at school are the most frequent reason for their initial referral for diagnostic evaluation. However, there is only little research examining the question whether the extra demands associated with ADHD impact negatively on teachers’ ratings of ADHD-diagnosed students and by this might handicap students from fulfilling their full academic potential. Greene et al. study (2002) showed that teachers’ reported levels of stress associated with supervising students with ADHD were a result of the “reciprocal process” of teachers’ and ADHD-diagnosed students’ individual characteristics. Comorbid levels of oppositional/aggressive behavior and/or social impairment represented the students’ characteristics that were identified to impact on teachers’ stress levels. ADHD behavior per se however had no significant impact. Biological age or teaching experience of teachers was unrelated to the teachers’ reported stress. Furthermore, individual differences between teachers’ perceived tension or frustration in response to ADHD behavior did not correlate with the actual levels of stress measured following real-life exposure to teaching students with ADHD (Greene et al. 2002). These findings might suggest that even if teachers disapprove verbally described ADHD behavior, actual contact to ADHD-diagnosed students still might impact on reported stress levels the other way round. Cornett-Ruiz and Hendricks (1993) showed that teachers’ initial negative impressions toward a videotaped child displaying stereotypical ADHD behavior had a negative impact on predictions concerning the child’s future academic success (i.e., likelihood of attending college or getting employed), independent of labeling the child to suffer from ADHD. However, teachers did not judge the performance on a hand-written essay of the ADHD-diagnosed child to be inferior to essays written by unaffected controls, showing that stigma associated with a diagnostic status influenced teachers’ professional attitude less than videotaped deviant behavior did (Cornett-Ruiz and Hendricks 1993). Recently, Bell et al. (2011) published a study examining the impact of teachers’ former training on the differences of teachers’ awareness of stigmatizing feelings in students that are suffering from ADHD. Accordingly, Bell et al. (2011) applied the ADHD Stigma Questionnaire of Kellison et al. (2010) to two groups of teachers differing on whether they had acquired a “special education certificate” in the past or not. This education is believed to prepare teachers for working with children that require special education. In line with the authors’ assumption, teachers with more knowledge about ADHD were more aware of possible stigmatizing perceptions of ADHD-diagnosed students. Whereas all teachers in this study supported the idea that students with ADHD worry about social consequences of disclosing their condition to others, teachers who obtained a “special education certificate” were disproportionally more inclined to suggest that students with ADHD actually experience social rejection due to their condition. Moreover, Bell et al. (2011) did not reveal any differences in scores on the ADHD Stigma Questionnaire (ASQ) due to the teachers’ age, gender, ethnicity, or years of experience as a teaching instructor. Although Stine (1994) highlighted appreciable contributions of learning instructors and educational staff in decreasing prejudices toward children with ADHD, Eisenberg and Schneider (2007) elaborated on the idea that the gender of the ADHD-diagnosed child might be crucial in determining the teacher’s evaluation of the child. Indeed, both teachers and parents have been found to show tendencies of rating girls with ADHD generally more negatively than their male counterparts (Eisenberg and Schneider 2007). These tendencies were even present after controlling for the child’s actual level of externalizing behavior. These observations are in accordance with previous findings indicating that ADHD may be less tolerated in girls by the environment (Hartmann 2003; Law et al. 2007). Moreover, Eisenberg and Schneider (2007) revealed a disproportional disagreement between the ratings of children with ADHD concerning their own school performance and objective school measures (math, reading, and language abilities), as compared to the estimates of healthy children. Interestingly, boys with ADHD were more negative toward their own mathematical skills, resulting in an underestimation of their apparent capacities. Negative self-perceptions were interpreted by Eisenberg and Schneider (2007) to reflect internalized stigmatizing expectancies the child acknowledged in the past by various sources of its environment such as teachers, parents, and peers. Given that only a minority of ADHD-diagnosed children (around 20 %) presents with learning deficits (Clarke 1997), the findings by Eisenberg and Schneider (2007) are of particular importance. It can be concluded that school staff predict academic achievements of children with ADHD to be worse than what has been actually confirmed by performances on standardized tests or the child’s degree of externalizing behavior. Tuchman (1996) emphasized that active sanctioning of disruptive behavior by teachers may increase the likelihood that children with ADHD get socially isolated and by this have to face enhanced stigmatizing attitudes of peers. As previously mentioned, negative attention by teachers is unlikely to restrict itself to the affected children with ADHD but is more likely to expand to the whole class environment (Whalen et al. 1981; Frederick and Olmi 1994). As ADHD cannot only result in a handicap during high-school years but also when academic demands increase with higher education, Vance and Weyandt (2008) assessed university and college professors’ conceptions about students with ADHD. Data analysis revealed that “years of experience,” “level of education,” “experience with college students with ADHD,” or “training in ADHD” had no significant effect on the professors’ cognitions concerning students diagnosed with ADHD. However, this study revealed that more than 40 % of professors did not agree on the idea that students with ADHD are equal to a learning disabled student. Nearly 50 % of the professors did not believe that ADHD-diagnosed students achieve lower average grades than not diagnosed students and slightly < 30 % were not in favor for additional academic support, such as providing copies of lecture notes or alternative assignments for students with ADHD. Less than a third of the respondents agreed on the idea that teaching ADHD students was more stressing. Even though Vance and Weyandt (2008) concluded that professors were generally informed about ADHD, the majority of professors acknowledged feelings of lacking information concerning ADHD and endeavored additional training in ADHD-related issues. It remains open to future research to investigate whether students with ADHD are aware and/or disadvantaged through school- or university teachers’ perceptions. It has to be kept in mind that denying access to resources (e.g., additional support in the learning environment) and/or undermining the disorder’s impact on everyday (school) life, can be a sign of stigma. This is of particular importance, considering the increasingly high numbers of students with learning disabilities including ADHD (Harris and Robertson 2001).

### Public stigmatization toward adults with ADHD

According to Burch (2004), ADHD in adulthood is even more likely than ADHD in childhood to be associated with misperceptions, confusion, and an increased number of laypeople and professionals lacking disorder-related knowledge. Burch (2004) further proposed that the subjectivity of diagnostic criteria might lead to public- as well as self-stigmatization of individuals diagnosed with ADHD. Schmitz et al. (2003) scanned media reports with regard to the etiology and treatment for ADHD in order to clarify which *social representations* of ADHD exist in the American population. In line with Burch (2004) assumption that public prejudices arise at least partly from the inconsistency and diversity of diagnostic criteria that have been applied over the last decades, Schmitz et al. (2003) found an association between changing DSM criteria and prototypical presentations of ADHD in lay people’s press. Discrepancies were found between lay people’s conceptions about ADHD treatment for choice and those recommended by professionals. Whereas professional articles nearly univocally supported the combined treatment for ADHD with behavioral therapy and medication, lay people were still indecisive how to proceed if asked to choose the most suitable treatment. However, Schmitz et al. analysis (2003) underlines that the stereotypical ADHD media profile can be best described as “a young white middle-class boy suffering foremost from hyperactivity.”

Throckmorton (2000) assessed attitudes of undiagnosed professionals working in the social sector, toward pictured colleagues suffering from a mental disorder. Responses of health professionals were scored with regard to their intrinsic levels of stigma concerning the colleague’s diagnostic status and competence as well as ratings of the colleagues’ ethical behavior related to the observation of staying in labor force after receiving a diagnosis of a mental disorder. In line with previous findings (Angermeyer and Dietrich 2006), professionals’ stigma levels differed markedly across pathologies, including schizophrenia, substance-related disorder, gambling, eating disorders, mood disorders, anxiety disorders, and ADHD. Interestingly, stigma scores associated with anxiety disorders were similar to those seen in response to a colleagues’ ADHD diagnosis. However, the colleague’s competence or ethical liability due to one of the different types of disorders was not questioned by respondents. This might indicate that a certain degree of stigma exists in health professionals and may depend on the nature of the disorder but at the same time does not need to affect the unaffected colleague’s expressed trust toward the professional qualification of a colleague with a psychiatric condition.

Jastrowski et al. (2007) measured the possible benefits of self-disclosing behavior of ADHD-diagnosed adolescents fictively depicted in vignettes. Vignettes differed with regard to ADHD symptoms (predominantly hyperactive vs. predominantly inattentive) and disclosure strategies (preventive disclosure or no signs of explicit disclosure at all). The authors showed that active disclosing behavior of the ADHD-diagnosed character significantly enhanced participants’ predictions concerning the fictitious peers’ likelihood of improving through means of treatment. Furthermore, approach behavior of participants was enhanced if the vignette’s character explicitly disclosed the condition by means of preventative disclosure strategies. Preventative disclosure is characterized by informing only those who actively noticed the adversities associated with ADHD symptoms and by being rather conservative in situations in which symptoms were not evident. Jastrowski et al. (2007) concluded that preventative disclosure can be an effective strategy in balancing out stigma associated with ADHD. They further verified stigma reducing effects of positive expectations of unaffected adolescents toward the efficacy of ADHD treatment. In order to identify the characteristics of undiagnosed individuals associated with an increased tendency to stigmatize individuals with ADHD, Canu et al. (2008) asked adolescents to indicate their likelihood to socially engage with a fictional male or female peer suffering from “clinically significant ADHD symptoms,” “a medical problem/impairment,” or “an ambivalent personality trait.” Fictitious individuals who were introduced to suffer from ADHD were univocally rated more negative compared to the two other vignettes. Furthermore, levels of agreeableness in male and female participants positively enhanced social acceptance of the target with ADHD diagnosis. Whereas this association was only present in female participants when the fictional target was of the same sex, male participants’ levels of agreeableness predicted social acceptance of the ADHD target independent of the target’s gender and/or outer appearance. Moreover, males scoring high on agreeableness did not only express greater social acceptance of someone with ADHD but were also more willing to socially interact with the vignette’s character in general. Female participants’ level of extraversion was shown to be the strongest predictor with regard to the expressed positive appraisal of the fictitious characters that was either of the same sex and diagnosed with ADHD or male and introduced to suffer from a medical problem. In contrast, extraverted boys were less inclined to engage with an ADHD-diagnosed female target; however, more introverted males expressed less pronounced negative attitudes toward a female character with ADHD than their extraverted counterparts. Interestingly, women, who scored high on conscientiousness, expressed less willingness to initiate social interactions and/or getting along with a male character with ADHD in general. In more detail, highly conscientious female participants expressed negative concerns about cooperating with a diagnosed male in academic and work-related settings. However, cooperation, working on a mutual goal and by this increasing the contact to members of stigmatized groups is one of the favored strategies in reducing stigma (Rüsch et al. 2005). Finally, in contrast to the authors` expectations, higher levels of openness and emotional intelligence were not found to predict higher appraisal scores toward peers with ADHD (Canu et al. 2008).

### Courtesy stigma

Reviewing the literature on courtesy stigma in ADHD revealed that objective measurement tools are lacking. It appears that courtesy stigma-related topics most often emerged rather automatically when parents sought out help by health professionals, researchers, and/or self-referred groups.

According to Goffman (1963), courtesy stigma predisposes an individual close to someone affected by stigma to get judged negatively as well, purely because of the individual’s association to the stigmatized person. Tuchman (1996) examined qualitative data obtained by interviewing parents of children with ADHD, children`s statements, and observations of interactions between children and their parents during self-referred group sessions. The results indicated that courtesy stigma is likely to arise from public`s discrediting attitudes toward parents for their child’s inability to fit into social norms. Mothers appeared to be particularly vulnerable to self-stigma. Statements by other parents, friends as well as family members let many mothers to engage in internalizing feelings of shame and self-accusation (Tuchman 1996). Moreover, stigmatization of parents can be found in diagnostic tools for ADHD. Tuchman (1996) referred in this context to Conrad (1978) who criticized questionnaires that are handed out to teachers and parents in order to specify whether a child meets ADHD criteria. According to Conrad (1978), often more weight is given to the teachers` ratings than to parent s` ratings during the final evaluation of those questionnaires within the diagnostic process.

Norvilitis and associates (2002) used discrepancies between the attitudes toward ADHD of parents of children without ADHD and the expectations of parents with children with ADHD to indicate the degree of perceived stigma of parents with children with ADHD. Results contradicted former studies showing heightened levels of depression in parents of children with ADHD (Johnston 1996; Byrne et al. 1998; West et al. 1999). Measures for depression, stress, perception of social support, and overall life satisfaction did not differ between the two parent groups. With regard to parental stigma perception, mothers of ADHD-diagnosed children stated more negative feedback concerning their parenting style than mothers of children without ADHD. Own parents or in-laws were, surprisingly, those who most frequently expressed critics concerning the mother’s capacity to parent the child with ADHD. The criticism by others has been found to have negative consequences on the mother’s well-being as reflected by moderate-to-large significant correlations between criticism on parenting style and both depression and perceived social support (Norvilitis et al. 2002). Evaluation of the Courtesy Stigma Questionnaire revealed further that mothers of the two groups did not differ in their attitudes toward ADHD in general. Even though mothers of ADHD children assumed that mothers with an unaffected child are holding negative ideas on about 75 % of the questionnaire’s items toward them, openly expressed attitudes on surveys or in direct discussions about ADHD-related issues by mothers of undiagnosed children were in general supportive and colored with sympathy (Norvilitis et al. 2002). Norvilitis et al. (2002) therefore proposed to educate mothers of children with ADHD about the actually positive beliefs of mothers with children without ADHD in order to alleviate the levels of internalized courtesy stigma. However, given the observation that mothers of children with ADHD were more often accused for bad parenthood by significant others (e.g., partners or other family members) than mothers of children without ADHD (Norvilitis et al. 2002; Koro-Ljungberg and Bussing 2009; Singh et al. 2010), interventions should also focus on family dynamics since they appear to be a crucial factor in mothers’ feelings of self-efficacy concerning their parenting style.

Harpur et al. (2008) found parental stigma to be positively associated with socio-cognitive costs linked to ADHD medication. Costs of ADHD medication were conceptualized to represent parents’ experienced adversities due to the child’s medication intake. Costs were represented by statements such as medication intake interferes with the child’s desire to initiate action, interferes with the child’s personality, and sets the child into a state of dizziness. The authors found that the higher the parental perceptions of public stigma, the higher the parent reported consistency in medication compliance, suggesting that stigma awareness in parents seems to be strongly related to doubts concerning stimulant medication but at the same time enhances rigidity in parents’ behavior adhering to treatment plans. Fathers scored significantly higher on levels of parental stigma, medication flexibility, and costs of medication treatment than mothers. This observation is supported by Tuchman (1996) who suggests that fathers display increased tendencies to blame themselves for their children’s condition giving their tendency to recognize themselves in their children’s misbehavior.

Koro-Ljungberg and Bussing (2009) evaluated possible factors influencing parental help-seeking behavior for their children’s ADHD symptoms in a community-based sample. Even though not initially attempted by the researchers, parental stigma perceptions emerged naturally through group discussions and were identified to impact on parental levels of distress. Moreover, parental levels of stigma were found to be related negatively to parents’ and children’s willingness to make use of community health programs. Furthermore, consequences of stigma varied in modality, depending on its originating source. If stigmatizing beliefs were put forward by partners or other members of the immediate family, experienced adversities seemed to be more pronounced for the parents’ self-esteem than if a broader community commented on their parenting. Accordingly, coping strategies are believed to be most effective if matched to the eliciting source from which feelings of parental stigma are arising from. Koro-Ljungberg and Bussing (2009) categorized parental coping strategies initiated to deal with stigmatizing attitudes of others to be diverse, ranging from simple denial of the child’s ADHD diagnosis to not disclosing the child’s diagnosis to even more radical and foremost emotionally driven actions such as calling the police to solve family affairs. In contrast, strategies intended to handle courtesy stigma within the family system are marked foremost by parents’ effort to provide as much guidance for their children with ADHD as possible. Koro-Ljungberg and Bussing (2009) interpreted these observations to be the consequences of the parents’ intention to reduce imagined and actually encountered public accusations of not fulfilling their parental role. Further parental actions for reducing stigmatizing attitudes extended to making their children’s homework, volunteering at school, running educational ADHD campaigns, and advocating special academic curriculums for their affected children. In this context, it is remarkable that a reoccurring theme in parents’ reports is the fact that parents have the impression to give disproportional less attention to their unaffected children.

Schmitz et al. (2003) call attention to the general disadvantage of groups with other than white middle-class backgrounds in the diagnostic assessment of ADHD. As diagnoses are foremost based on inventory cut-off scores of white middle-class reference samples, it seems crucial to question how norm scores generalize to other ethnical groups (Schmitz et al. 2003). By making use of ADHD as a model disorder known for its association with stigmatizing prejudices, Kendall and Hatton (2002) found that the American public is more likely to accuse ADHD symptoms in African–American to stem from poor parenthood than in white Americans. By this, parental constraints due to stigma are increased in already disadvantaged groups.

dosReis et al. (2010) acknowledged the general overrepresentation of studies, media reports, and scientific concerns for ADHD in an audience of white middle-class citizen and demonstrated that courtesy stigma can be objectified across different ethnicities. Accordingly, comparable signs of parental stigma associated with ADHD were found in a group of socio-economical disadvantaged African–Americans (Mychailyszyn et al. 2008). dosReis et al. (2010) further showed that the likelihood of stigma associated with ADHD to shape parental attitudes toward ADHD and its treatment is comparable in ethnicities other than white Americans. The majority of their participants were African–American urban citizens of low-income households, with most of them rearing a son with a diagnosis of ADHD. Similar to the concerns of white middle-class participants of former studies, the majority of respondents expressed fears about others rating their children’s ADHD to be a consequence of bad parenting, inadequate disciplining, and lack of structure. These maladaptive cognitions were again interpreted as consequences of courtesy stigma (dosReis et al. 2010). Moreover, nearly half of the parents (44 %) felt uncomfortable when reflecting on the possibility that others might belief that they seek out professional help just to get extra benefits from social institutions or schools. Furthermore, social withdrawal from significant others and the children’s peers was familiar to nearly half of the respondents (40 %), which resulted in feelings of social deprivation. Bullying of their child was reported frequently, leading parents to fear that ADHD-related peer rejection will adversely impact on children`s self-worth. Moreover, constantly encountering public’s negative reactions to their children’s behavior and educational as well as social failure caused some of the parents ruminating about whether they are allowed to talk as positive about their children’s accomplishments as other parents do. Parental perceptions of faint and capitulation were linked to feeling helpless in convincing others how it is to raise a child with ADHD. Twenty-one percent of the parents reported feeling misunderstood by teachers or primary medical professionals, and 6 % of parents mentioned being exposed to negative renunciation by significant others. These accusations led them consider whether they should end their children’s prescribed medication (dosReis et al. 2010).

## Conclusion

The present paper reviewed empirical knowledge regarding stigma associated with ADHD. ADHD is known for its lifetime persistency and is characterized by clinical symptoms of inattention, impulsivity, and hyperactivity (American Psychiatric Association 1994), showing its impact on many facets of patients’ life. Moreover, patients are at high risk for additional cognitive (Dowson et al. 2004; Dige and Wik, 2005; Faraone et al. 2006) and social impairments (Guevremont and Dumas 1994; Mannuzza and Klein 2000), as for example seen in fewer social acquaintances, difficulties in intimate relationships, and general deviance in social adjustment (Hechtman 2000; Semrud-Clikeman 2010). It has been suggested that varying degrees of symptom presentation (predominantly hyperactive vs. predominantly inattentive) and variation of symptom severity across temporal and contextual situations increases the risk of questioning the disorders’ reliability (Burch 2004; Koro-Ljungberg and Bussing 2009). Diversity in the disorder’s etiology as well as the disorder’s heterogeneity across age groups (Burch 2004) have been shown to enhance the disorder’s proneness to stigma, partly through questioning the disorders’ diagnosis, assessment, and treatment (Stine 1994; Clarke 1997; Schmitz et al. 2003; Pescosolido et al. 2007; Harpur et al. 2008; Davis-Berman and Pestello 2010; dosReis et al. 2010). As public awareness increases concerning the disorder’s associated ambiguity, it is likely that diagnosed individuals are becoming the focus of stigmatizing cognitions of undiagnosed social accompanists and/or patients with psychiatric conditions itself (Harpur et al. 2008; Coleman et al. 2009; Kellison et al. 2010). The impact of media in strengthening misperceptions and stigmatizing beliefs about patients suffering from ADHD was stressed (Slopen et al. 2007) and can be seen as a likely source open to be challenged through public education implementing information about stigma and ways how to prevent it. Additional information about ADHD etiology has been shown to help people close to the affected individual to antagonize public stigma (Liffick 1999; Throckmorton 2000; Biederman and Faraone 2004). Likewise, informing the public about existing stigmatizing dynamics might serve the superordinate goal of lessening patient’s burdens caused by stigma (Rüsch et al. 2005; Corrigan and Shapiro 2010). Empirical data of the discussed studies nearly unequivocally stressed the idea that stigma related to ADHD affects treatment adherence and treatment efficacy adversely (Stine 1994; Burch 2004; Harpur et al. 2008) by lowering the individual’s self-esteem and or levels of self-efficacy (Rüsch et al. 2005). Moreover, symptom aggravation in response to internalized self-stigma and anticipated public’s degradation was mentioned (Burch 2004). Whereas some studies supported the idea that neither diagnostic labeling itself (Cornett-Ruiz and Hendricks 1993; Law et al. 2007; Sandberg 2008) nor medicational disclosure (DeSantis et al. 2008; Sandberg 2008; Davis-Berman and Pestello 2010; Singh et al. 2010) elicit greater stigmatizing tendencies of undiagnosed fellows, negative effects were found for actual exposure of healthy participants to ADHD symptom presentation (Cornett-Ruiz and Hendricks 1993; Norvilitis et al. 2002; Kendall and Shelton 2003; Hoza et al. 2005; Singh et al. 2010). Exposure of individuals without ADHD to ADHD-associated behavior (e.g., as presented on videotapes) increased the tendency in the individual to rate the presented person less favorably.

However, recently emerging studies investigating the effects on stigma reduction of co-operational behavior of children with and without ADHD diagnosis are very promising. For example, Sandberg (2008) showed that boys with ADHD were appreciated by their peers despite them being aware of the diagnostic status. Perceived dangerousness of the diagnosed person was a reoccurring theme in both the literature on adult as well as childhood ADHD, which had an impact on stigmatizing prejudices toward ADHD (Slopen et al. 2007; Pescosolido et al. 2007; Walker et al. 2008). This perceived dangerousness was most often described to arise from heightened levels of externalizing behavior associated with the patient’s diagnostic status and the public’s expressed aversion to get in contact with individuals diagnosed with ADHD. Corrigan and Shapiro (2010) however stressed the importance of getting in contact with members of stigmatized groups in order to reduce negative affect and by this stigma toward the individual. Moreover, many studies acknowledged that the public is handling different moralistic frameworks when rating disorder seriousness and disorder reliability with regard to ADHD versus medical conditions, such as asthma (Liffick 1999; Martin et al. 2007; Pescosolido et al. 2008). However, the aim of future studies should be to evaluate to what extent ratings on self-administered questionnaires reflect ecologically valid attitudes toward individuals with ADHD. Since most of the studies made use of vignettes depicting characters suffering from various conditions, followed by questionnaires which measure the respondents’ willingness to approach an individual with ADHD, one might question whether attitudes measured by self-administered paper–pen assessments are sensitive enough to predict actual behavior that can be disadvantageous for some but not for others. This applies also to our knowledge on stigma related to authorities, in particular to teachers, which is mainly based on self-rated assessment. Observational studies in the class environment including the class dynamics might support to objectify the qualities affecting teacher–student interactions. In line with this, Hebl and Dovidio (2005) recommended research paradigms that focus on actual social interactions between stigmatized targets and stigmatizing offenders in order to assess the impact stigma may exert on the daily functioning of the discriminated person. When assessing signs of self-stigma due to ADHD diagnosis, objective measures of the stigmatized individuals (e.g., the frequency of missed school attendance) and the individuals’ levels of stigma perception might be matched in order to objectify consequences of stigma on the individuals’ daily life. Accordingly, Kellison et al. (2010) psychometrically validated their ADHD Stigma Questionnaire (ASQ), which can be used to estimate a respondent’s stigma perception with regard to ADHD, independent of the respondent’s health/psychiatric status. The finding that the ASQ can be used in populations other than individuals with ADHD is promising (Bell et al. 2011). Further shortcomings of the reviewed literature are that many studies were of correlational nature or represented inventories of anecdotal statements of individuals participating in self-help groups. Furthermore, studies focusing on the effect of stigma on siblings of children diagnosed with ADHD are missing. Literature on stigma toward adults with ADHD is also rather occasional. Overall, it appears that current knowledge on stigma in ADHD is largely based on opinions of respondents who experienced stigmatization associated with their own or their relatives’ ADHD. Consequently, these individuals might have been highly motivated to participate in studies. However, this might also limit the representativeness of current knowledge with regard to the general population or the total of patients with ADHD and their relatives. Furthermore, studies on ADHD in adulthood primarily examined students from undergraduate programs, which again limit the representativeness of results. It has also to be pointed out that there is an overrepresentation of white middle-class participants in studies concerning stigma in ADHD (Kendall and Hatton 2002; Mychailyszyn et al. 2008; dosReis et al. 2010) raising the demand for more ADHD stigma-related research across different ethnicities. Finally, as Rüsch et al. (2005) discussed, stigma might only be detrimental to individuals if they identify themselves with the target group of stigma. In this respect, future research evaluating the effects of stigma on individuals with ADHD should take personality characteristics into consideration, such as the extent to which individuals associate themselves with the disorder and are sensitive to negative cognitions of others. This is of particular interest, since knowledge concerning illness awareness in individuals with ADHD is very limited (Burch 2004; Weisler and Goodman 2008).
